# Brain region-specific effects of nearly fixed sapiens-derived alleles

**DOI:** 10.1186/s12863-022-01048-8

**Published:** 2022-05-11

**Authors:** Alejandro Andirkó, Cedric Boeckx

**Affiliations:** 1 University of Barcelona, Barcelona, Spain; 2 University of Barcelona Institute of Complex Systems, Barcelona, Spain; 3 ICREA, Barcelona, Spain

**Keywords:** Human evolution, Brain, cis-eQTL, Gene regulation

## Abstract

The availability of high-coverage genomes of our extinct relatives, the Neanderthals and Denisovans, and the emergence of large, tissue-specific databases of modern human genetic variation, offer the possibility of probing the effects of modern-derived alleles in specific tissues, such as the brain, and its specific regions. While previous research has explored the effects of introgressed variants in gene expression, the effects of *Homo sapiens*-specific gene expression variability are still understudied. Here we identify derived, *Homo sapiens*-specific high-frequency (≥90*%*) alleles that are associated with differential gene expression across 15 brain structures derived from the GTEx database. We show that regulation by these derived variants targets regions under positive selection more often than expected by chance, and that high-frequency derived alleles lie in functional categories related to transcriptional regulation. Our results highlight the role of these variants in gene regulation in specific regions like the cerebellum and pituitary.

## Significance statement

We show that almost-fixed variants distinguishing *Homo sapiens* from Neanderthals and Denisovans have a previously underexplored role in the evolutionary history of brain regions. We present evidence that these variants accumulate in genomic regions under positive selection, and that correlation with brain volume GWAS top hits, suggesting a role of genetic regulation in shaping tissues such as the cerebellum.

## Introduction

Geometric morphometric analysis on endocasts [1–5] have revealed significant differences between Neanderthal and *Homo sapiens* skulls that are most likely the result of differential growth of neural tissue. Specific brain regions such as the cerebellum, the parietal and temporal lobes have been hypothesized to have expanded in the *Homo sapiens* lineage, with potential consequences for the evolution and diversification of cognitive skills. Probing the nature of these consequences is challenging, but the availability of several high-quality Neanderthal and Denisovan genomes [6–9] has opened numerous research opportunities for studying the evolution of the *Homo sapiens* brain with unprecedented precision.

Efforts have been made to determine the molecular basis of species differences based on a small number of fixed missense mutations that are *Homo sapiens*-specific [10, 11]. However, evidence is rapidly emerging in favor of an important evolutionary role of regulatory variants, as originally proposed more than four decades ago [12]. For instance, regulatory variants are overrepresented in selective sweep scans to detect areas of the genome that have been significantly affected by natural selection after the split with Neanderthals/Denisovans [13].

The increasingly important role of gene regulation in the evolution of *Homo sapiens* has led to the idea of connecting vast datasets of variation in genomic regulation to the genetic sequences obtained from extinct humans. For example, a major study [14] explored the effects of Neanderthal and Denisovan introgressed variants in 44 tissues and found downregulaton by introgressed alleles in the brain, particularly in the cerebellum and the striatum. In a similar vein, another study [15] examined the effects of extinct human introgression on brain and skull shape variability in a modern human population to determine which variants are associated with the globularized brain and skull that is characteristic of our lineage. In consonance with [14], the variants with the most salient effects were those found to affect the structure of the cerebellum and the striatum. Crucially, for these questions to be asked, we must move beyond fully-fixed variants, and embrace the variation found within modern human populations.

Building on these efforts, we decided to relate derived, modern-specific alleles found at very high frequency across modern populations to gene expression in the brain, in order to examine the effects of genetic variation relative to Neanderthals and Denisovans. To this end, we took advantage of a recent systematic review, [16], which provides an exhaustive dataset of derived, *Homo sapiens*-specific alleles in modern human populations. This dataset includes a subset of nearly-fixed (≥90*%*) variants that can determine common trends in current human populations compared to other extinct human species.

To determine the predicted effect on gene expression of these alleles we exploited the GTEx database. The GTEx data consist of statistically significant allele effects on gene expression dosage in single tissues, obtained from tissues of adult individuals aged 20 to 60 [17]. By offering information about Expression Quantitative Trait Loci (cis-eQTLs) across tissues, the GTEx database forces us to think beyond variants that affect the structure and function of proteins, as well as to consider those that regulate gene expression.

While the important role genetic regulation in human evolution has been highlighted by previous studies [18–21], we find that species-specific variants above a high frequency threshold have a previously underexplored role in human brain evolution. We show that regions under putative positive selection are enriched in derived, high-frequency (HF) eQTLs, and that the pituitary and cerebellum have a significantly higher number of regulatory variability compared to other tissues and a control set. We also show that derived alleles tend to have a downregulating effect but only when linkage disequilibrium is not controlled for, a result that contrasts with previous research on introgressed variants [14]. Finally, we present a two sample Mendelian randomization analysis that correlates variability in genes related to neurodevelopment and brain volume GWASs.

## Results

We retrieved variation data from [16], a dataset that determines *Homo sapiens* allele specificity using three high-coverage archaic human genomes available at the moment (the Altai and Vindija Neanderthals [6, 7], and a Denisovan individual [8]).

The variation data was crossed with the list of variants obtained with the GTEx significant cis-eQTL variants dataset to determine if the selected variants affect gene expression, focusing on 15 central nervous system-related tissues. The GTEx data consist of statistically significant allele effects on gene expression dosage in single tissues, obtained from brain samples of adult individuals aged 20 to 60 [17]. The resulting dataset is composed of *Homo sapiens* derived alleles at high frequency that have a statistically significant effect (at a FDR threshold of 0.05, as defined by the GTEx consortium [22]) on gene expression in any of the selected adult human tissues.

### Functional categories and tissue-specificity

In quantitative terms, our data amounts to 8,271 statistically significant SNPs associated with the regulation of a total of 896 eGenes (i.e., genes affected by cis-regulation). When controlling for total eQTL variance between brain regions, a Chi-square test reveals that the proportion of derived, HF eQTLs across tissues is significantly different compared to the rest of non-derived, non-high-frequency eQTLs (*p*<2.2*e*−16). A post-hoc residual analysis indicates that regions such as the pituitary and the cerebellum are among the major contributors to reject the null hypothesis that the distribution is similar between both groups (*p*<0.05). In other words, the pituitary and the cerebellum are the two brain regions where *Homo sapiens*-specific eQTLs accumulate relative to the control set of variants.

Derived eQTLs at high frequency are significantly different from the categories of the rest of GTEx eQTL variants in brain tissues (Chi-square test, *p*<2.2*e*−16). NMD (nonsense-mediated mRNA decay target) transcript, non coding transcript, and 5 ^′^-UTR (untranslated region) variants are the categories driving significance (*p*=<2.2*e*−16 for the three sets, residual analysis).

**Fig. 1:**
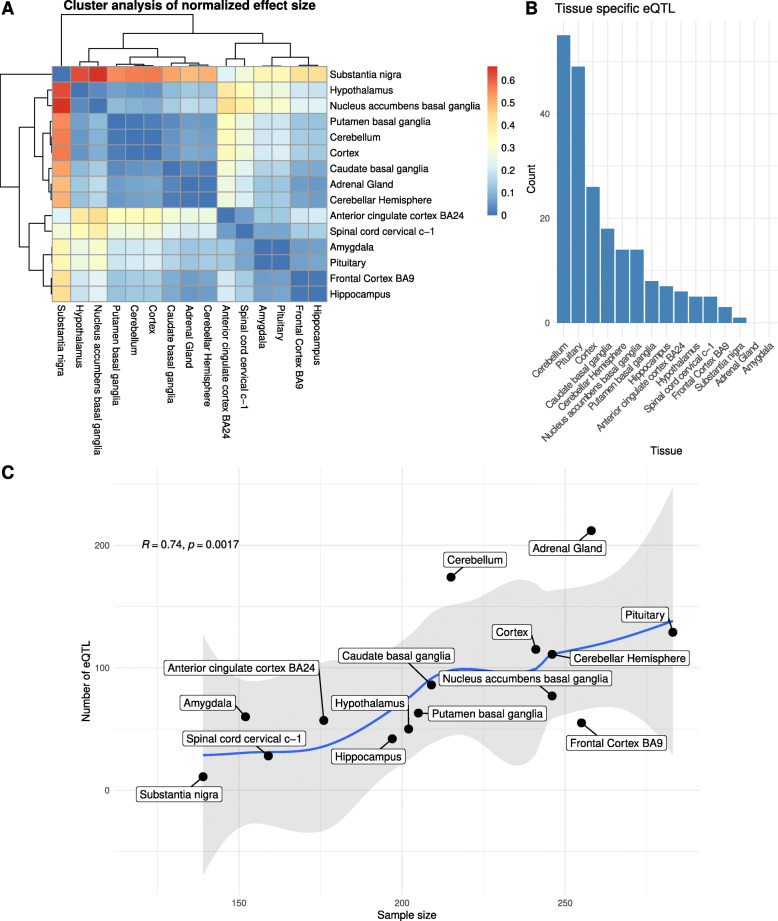
**A** Hierarchical clustering analysis of eQTL normal effect size, not controlled for linkage disequilibrium (LD). Color denotes hierarchical distance. **B** Number of tissue-specific eQTLs after clumping. Adrenal gland and Amygdala do not contain tissue-specific eQTL in our dataset. **C** Brain region sample size and eQTL count correlate in our dataset. The blue line marks a polynomial regression line fit, with regression’s standard error confidence intervals (95%) in gray

To account for linkage disequilibrium and ensure statistical independence, variant clumping was applied through the eQTL mapping p-value at a *r*2=0.1. After clumping, the dataset was reduced to 1,270 alleles across tissues, out of which 211 are region-specific (Fig. 1B). Because eQTL discovery is highly dependent on the number of tissue samples [22], tissues with more samples tend to yield a higher number of significant variants, regardless of tissue specificity (Fig. 1C), as shown by a Spearman correlation test (*p*=0.0017; *r*=0.74, controlled for linkage disequilibrium). A polynomial regression line fit (blue line in Fig. 1C) shows that the cerebellum, adrenal gland and BA9 fall outside the local regression’s standard error confidence intervals (in gray in Fig. 1C).

We sought to understand if the cerebellum, adrenal gland and BA9 stand out considering that most eQTLs are shared among regions. The distribution of clumped region-specific variants (Fig. 1B) does not correlate with GTEx RNAseq sample size (*p*=0.9495, Pearson correlation test). This lack of correlation might be explained by known effects of genetic regulation disparity between brain regions, reflected in distinct eQTL mappings for cerebellar tissue [23, 24]. Additionally, we designed a random sampling testing approach (*n*=100) to see if any particular region tends to draw more clumped unique eQTLs regardless of total eQTL values. The test reveals no significant difference in proportions (*p*=0.3647, Chi-square independence test). The fact that the adrenal gland and the amygdala have no unique clumped variants might be underlying this result.

### Genomic regions under positive selection are enriched in eQTLs

To determine further the evolutionary significance of any of the variants in our data, we ran two randomization and permutation tests (*N*=1,000) to test whether the derived HF eQTLs fell within regions under putative positive selection relative to other hominins as identified in two selective sweep studies [13, 25].

**Fig. 2:**
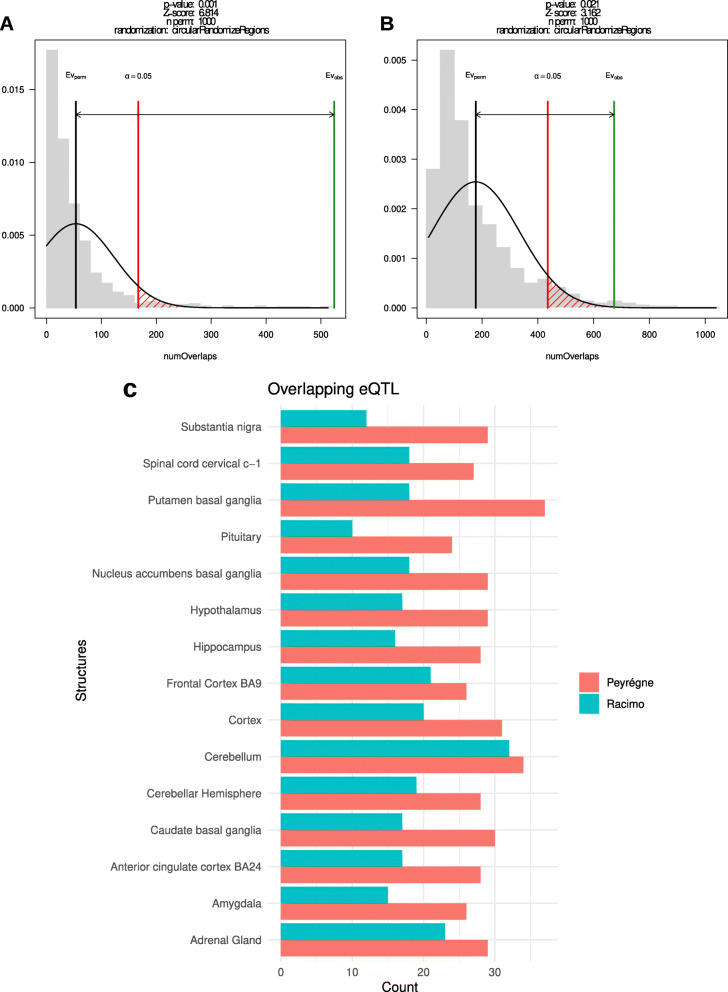
Derived, HF eQTLs are present more than expected by chance in selective sweeps from [13] (**A**) and [25] (**B**). **C** shows the count of eQTL overlapping with regions under putative positive selection per region

We found a significant (*p*=0.001, observed = 525 overlapping regions, expected = 53) overlap between eQTLs and regions of positive selection as defined by [13], as well as in an earlier independent study [25] (*p*<0.02, observed = 673, expected = 177, Fig. 2A and B). A Wilcoxon signed-rank test shows that the number of eQTLs found in positive selection regions (visualized per region in Fig. 2C) is significantly different between studies (*p*=6.104*e*−05, after controlling for length differences in the windows detected by each study). A Dunn test (after Bonferroni group correction) failed to find a significant difference between the count of alleles per region in each selective sweep, despite the apparent concordance of the studies in the cerebellum (Fig. 2C). We take this to mean that positive selection does not reflect a significant accumulation of eQTL variants in any given brain region, but rather seems to affect high-frequency derived eQTLs in general.

### eQTL directionality depends on LD but not allele frequency or brain region

A previous study [14] had suggested that Neanderthal alleles present in the modern human genetic pool downregulate gene expression in brain tissue. This study also used the GTEx data, but focused on Neanderthal introgressed variants as opposed to *Homo sapiens*-derived ones.

**Fig. 3:**
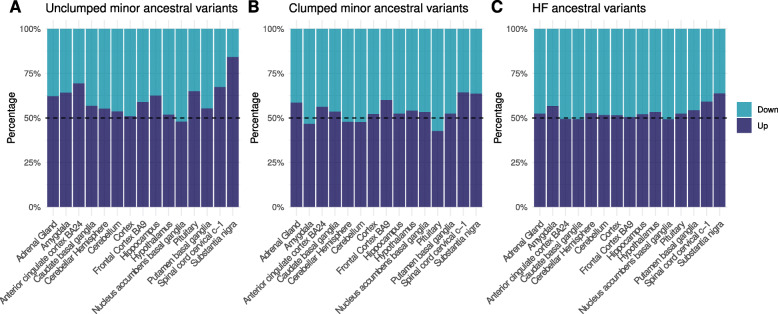
Distribution of up and down-regulating ancestral variants across different subsets of the data, in all eGenes. We include here data before (**A**) and after (**B**) controlling for linkage disequilibrium in minor alleles (≥10% frequency). A control using major ancestral alleles (at ≥90% frequency) is included (**C**)

In our derived HF eQTL dataset (Fig. 3B), we did not observe any significant deviance from the expected 50% proportion between down and upregulating variants (*p*=0.3656, Chi-square test). A significant deviance from the expected 50% proportion (*p*<2.2*e*−16, Chi-square test) does obtain, however, when linkage disequilibrium is not controlled for (Fig. 3A). A hierarchical cluster analysis of the distance of normalized effect size between regions in non-clumped eQTLs shows how the substantia nigra is particularly affected by the downregulating direction skewness effect (Fig. 1A). This contrasts with the result found by [14], who found this downregulation effect in cerebellum and the striatum in introgressed dataset, suggesting that variants specific to our lineage do not affect gene expression in the brain in a particular direction.

The same deviation from the expected 50% up and down-regulation proportion was present in major ancestral alleles at a 90% frequency threshold (*p*=<2.2*e*−16, Chi-square test, Fig. 3C), discarding the possibility that the asymmetry is due to allele frequency cutoffs. Post-hoc residual analysis shows that downregulating eQTL skewness affects different tissues in the major and minor ancestral eQTL sets. We conclude that asymmetric directionality of eQTL regulation is not specific to a given tissue nor is accounted for by frequency.

### Derived eQTLs are correlated with top hits in brain volume GWASs

As [14] had found that some of the introgressed variants from Neanderthals were also top GWAS hits, we hypothesized that derived variants might also reflect some of the changes that are characteristic of our species. We decided to focus on structural changes beyond the cerebral cortex since these are much harder to capture by endocasts, and they tend to be underrepresented in the brain evolution literature. By contrast, allelic effect in gene expression can be contrasted with modern brain volume GWAS studies via two sample Mendelian randomization tests. Thus, we chose 10 brain volume GWASs that are part of the UKBiobank and IEU GWAS curated catalogs. We selected four studies centered on the volume of distinct subregions of the cerebellum (left and right white matter tracts and cortices), as well as GWASs studying the volume of other subcortical structures: putamen, hippocampus, amygdala, thalamus, caudate and hippocampus (see Methods).

We first selected the top eQTL hit per gene and structure based on their eQTL p-value, under the assumption that is the variant more strongly associated with genetic regulation, and filtered by presence in the catalog of derived alleles by [16]. We chose not to use high-frequency variants exclusively, as pleiotropy and linkage disequilibrium may confound the results. Under a pleiotropy model, a variant affects two different phenotypes, mixing the signal of different GWASs, while linkage disequilibrium can affect two sample Mendelian randomization by falsely detecting causality in a high frequency variant that is only in high LD with the real causal variant (one not necessarily being almost fixed or derived). The selected variants were analyzed following Wald ratio tests per gene/structure volume associations.

The results (corrected by Bonferroni) highlight genes associated with neurodevelopment and cerebellar disorders. This is consistent with the kind of phenotypes one would expect for genes associated with brain volume GWASs. However, the importance of these results lies on pinpointing which specific genes have been affected over the course of *Homo sapiens* evolution. Among the genes related to cerebellar volume in the four substructure GWASs we find genes related to ataxia (*PEX7*, *MRPS27*, *PTK2* [26–28]), neurodevelopment (*YPEL3*, *CASP6*, *TRIM11*, *GNB5* [29–32]) and microcephaly (*PDCD6IP*, *USP28* [33, 34]). Of note, hits for other brain structures did not correspond with eQTL regulation in the relevant tissue or have no identified functional role in brain development.

To reveal if the eQTL signal was the same as those of brain volume GWAS top hits, we ran Bayesian colocalization tests for all the eQTL that survived two sample Mendelian Randomization. However, we found that the probability that GWASs and derived eQTLs share the same signal is very low (<6*%*). We therefore conclude that there is no causal relationship between eQTL expression changes and subcortical volume GWASs, and that the relationship identified here is of correlation.

## Discussion

In this study we sought to shed light on the impact of modern-human-specific alleles found at high frequency on gene regulation across brain regions. Our intention was to complement previous work that focused on the effects of introgressed variants from Neanderthals [14, 15].

We found that high-frequency derived eQTL indeed constitute a very useful category to understand phenotypical changes specific to our lineage. As reported in the results, these variants accumulate more than expected relative to the control set of eQTLs in the cerebellum and pituitary, are functionally differentiated and overrepresented in windows of the genome associated with signals of positive selection. Also, the enrichment of 5 ^′^UTR categories in HF derived eQTLs suggests a role for regulatory variants in *Homo sapiens* evolution (as discussed in [18–20]).

Contrary to [14] we did not find a significant skewness towards downregulation in derived eQTLs, regardless of frequency. This downregulating effect was previously detected as a characteristic of Neanderthal alleles introgressed in the modern human genetic pool [14]. The derived eQTLs examined here did show directional regulatory asymmetry but only when linkage disequilibrium was not controlled for. Additional testing indicates that the effect is not introduced by the high frequency cutoff imposed to the data, nor introduced by the bias of a particular region in either HF or non-HF alleles. We suggest that derived HF variants mapped as eQTLs might affect the modern human genetic regulation landscape in virtue of either being drivers of positive selection or being in linkage disequilibrium with causal, positively selected variants.

This idea is reinforced by our results in GWAS colocalization, showing that despite the correlation of eQTLs with subcortical brain volume GWAS top hits, there is no shared genomic signal between GWAS summary data and derived variants affecting gene expression variability. Several reasons could be put forward for this: It could be the case that the underlying causal variants are in high LD with derived eQTL and either (i) derived variants not captured by eQTL mapping, or (ii) non-derived variants that gain functionality by the effects of derived alleles in gene expression. Even if colocalization didn’t detect causal variants, some of the eQTLs correlated with GWAS hits might be affecting neural phenotypes that do not leave a clear imprint in endocasts. For example, we find that derived variability in genes related to cerebellar development is correlated with this substructure’s volume. The same effect was not found in other subcortical structures, as discussed in Derived eQTLs are correlated with top hits in brain volume GWASs section. However, the pituitary, along with the cerebellum, has a significantly high number of derived eQTLs relative to controls, not explained by LD artifacts (Fig. 2B). This is relevant in light of claims that the Hypothalamic-pituitary-adrenal (HPA) axis played a role in the evolution of our social cognition [35, 36].

We wish to stress that our focus on brain(-related) structures in no way is intended to claim that only the brain is the most salient locus of difference between moderns and Neanderthals/Denisovans. While other organs undoubtedly display derived characteristics, we have concentrated on the brain here because our primary interest lies in cognition and behavior, which is most directly affected by brain-related changes. In addition, we want to end with listing several limitations. First, like other current work making use of DNA retrieved from extinct hominins, we are constrained by the small number of high-coverage genomes currently available. While we certainly hope that this number will increase in the future, and yield a richer picture of variation in our relatives, it seems to us that despite this limitation, comparisons between us and our closest extinct relatives in the last decade have yielded valuable information that would not have been accessible otherwise. Second, our work would benefit enormously from an even better grasp of variation within human populations, and we look forward to more inclusive samplings in the future. Third, as indicated above, the GTEx dataset we used offers data from individuals aged 20 to 60 years. As such, it limits our ability to probe the nature of differential effects of derived alleles at earlier developmental stages, which are no doubt extremely relevant for all the brain regions examined here. Our findings will therefore have to be complemented with other methods to offer a more comprehensive view of recent brain evolution in the future.

## Methods

We accessed the *Homo sapiens* variant annotation data from [16]. The full dataset at the basis of this study is publicly available at 10.6084/m9.figshare.8184038. The catalog consists of archaic-specific variants as well as all loci displaying variation within modern populations, using the 1000 genomes project and ExAc data to determine frequencies and the human genome version *hg19* as reference. As described in the original article, the authors additionally imposed quality filters pertaining to the archaic genomes: sites with less 5-fold coverage and more than 105-fold coverage for the Altai individual, or 75-fold coverage for the rest of archaic individuals were not taken into consideration). For ambiguous cases, ancestrality of the relevant variant was assigned using multiple genome aligments [37] and the macaque reference sequence (*rheMac3*) [38].

For replication purposes, we wrote a script that reproduces the 90% frequency cutoff point used in the original study. We filtered the variants according to the guidelines in [16] such that: 1) all variants show 90% allele frequency, 2) the major allele present in *Homo sapiens* is derived. Ancestrality relative to great apes is either determined by the criteria in [37] or by the macaque reference allele in ambiguous loci. Ancestrality relative to extinct human species relies on two possible conditions: 1) either archaic reliable genotypes have the ancestral allele, or 2) the Denisovan carries the ancestral allele and one of the Neanderthals the derived allele (accounting for gene flow from *Homo sapiens* to Neanderthal).

Additionally, the original study we relied on [16] applies the 90% frequency cutoff point in a global manner: it requires that the global frequency of an allele be more than or equal to 90%, allowing for specific populations to display lower frequencies. Using the metapopulation frequency information provided in the original study, itself derived from the 1000 Genomes Project, we applied a more stringent filter and removed any alleles that where below 90% in any of the five major metapopulations included (African, American, East Asian, European, South Asian). We then harmonized and mapped the high-frequency variants to the data provided by the GTEx database [22]. In order to do so we pruned out the alleles that did not have an assigned rsIDs.

GTEx offers data for the following tissues of interest: Adrenal Gland, Amygdala, Caudate, Brodmann Area (BA) 9, BA24, Cerebellum, Cerebellar Hemisphere, Cortex, Hippocampus, Hypothalamus, Nucleus Accumbens, Pituitary, Putamen, Spinal Cord, and Substantia Nigra. Of these samples, cerebellar hemisphere and the cerebellum, as well as cortex and BA9, are to be treated as duplicates [17]. Although not a brain tissue *per se*, the Adrenal Gland was included due to its role in the Hypothalamic-pituitary-adrenal (HPA) axis, an important regulator of the neuroendocrine system that affects behavior.

Post-mostem mRNA degradation affects the number of discovered eQTLs in other tissues. However, we did not control for post-mortem RNA degradation, since the Central Nervous System has been shown to be relatively resistant to this effect [39]. However, re-sampled tissues (here labeled ‘cerebellar hemisphere’ and ‘Cortex’ following the original GTEx Consortium denominations) do show differences compared to their original samples (‘cerebellum’ and ‘BA 9’). We acknowledge that the resulting data are limited by inherent problems of the GTEx database, such the use of the same individuals for different brain tissue samples, the reduced discovery power of rare variants [17], and other artifacts introduced during RNAseq analysis.

Clumping of the variants to control for Linkage Disequilibrium was done with Plink (version 1.9) through the *ieugwasr* R package [40], requiring a linkage disequilibrium score of 0.90 (i.e., co-inheritance in 90% of cases) for an SNP to be clumped. The nominal p-value of eQTL mapping was used as the criterion to define a top variant; i.e., haplotypes were clumped around the most robust eQTL candidate variant. Linkage disequilibrium values are extracted from the 1000 Genomes project ftp server (ftp://ftp.1000genomes.ebi.ac.uk/vol1/ftp/release/ 20130502/) by the *ieugwasr* R package.

Distance values for tissue hierarchical clustering were calculated by using the mean values of the normalized effect size of derived HF eQTLs.

We performed the permutation test (n=1,000) with the R package RegioneR [41] using the unclumped data, as variants might clump around an eQTL falling outside windows of putative positive selection, underepresenting the number of data points inside such genomic areas and reducing statistical power.

We ran the two sample Mendelian Randomization tests at a *p*=5*e*−04 threshold for top hit identification through the *ieugwasr* [40], *MRinstruments*, and the colocalization tests through the *gwasglue* package. The selected GWASs for colocalization can be consulted in the relevant section of the article’s code.

Figures were created with the ggplot2 R package [42] and RegioneR [41]. All statistical tests were controlled for power (≥0.8). The human selective sweep data was extracted from Supplementary Table S5 of [25], and from Supplementary Table S2 of [13]. GWAS summary data and harmonized top eQTL instruments for two sample Mendelian Randomization were extracted from the IEU GWAS database API [40].

## Data Availability

The datasets supporting the conclusions of this article can be reproduced from the article’s Github code repository at https://github.com/AGMAndirko/GTEX-code. The original eQTL data can be retrieved from [17], the [16]. The GWAS summary data was retrieved through the *ieugwasr* package repository [40].
